# Mechanism and implications of pro-nature physical activity in antagonizing psychological stress: the key role of microbial-gut-brain axis

**DOI:** 10.3389/fpsyg.2023.1143827

**Published:** 2023-07-25

**Authors:** Hu Lou, Xue Liu, Ping Liu

**Affiliations:** School of Sports Science, Nantong University, Nantong, Jiangsu, China

**Keywords:** microbiota-gut-brain, psychological stress, pro-nature, physical activity, mechanisms

## Abstract

Appropriate physical activities and a biodiversity-rich environment are conducive to the relief of psychological stress, and pro-nature physical activities are a combination of the two, which has good application potential in antagonizing psychological stress, but the intervention mechanism is still unclear. The microbiota-gut-brain axis is cyclically associated with psychological stress, and psychological stress can affect the microbiota through the gut-brain pathway, and conversely, the microbiota can also affect the psychological stress-induced symptoms. It is suggested that the microbe-gut-brain axis may provide a new perspective and target for the treatment of psychological stress-related diseases. Pro-nature physical activity can improve the number of Firmicutes, short-chain fatty acids, Akkermansia bacteria, and the gut-brain barrier and further affect the HPA axis, BDNF, and serotonin pathways of gut-brain two-way communication, thereby maintaining the body’s homeostasis and reducing antagonistic psychological stress. According to the comprehensive influence of physical activities on the microbiota-gut-brain axis, a “green + exercise prescription hypothesis” in line with the holistic medical concept is revealed, which is expected to be effective in the prevention, alleviation, and treatment of irritable bowel syndrome and neurodegenerative diseases. It provides new means for treating psychological stress-related diseases such as mental disorders and mood disorders. In addition, it enlightens the construction of green infrastructure that is conducive to the diversified contact of microorganisms in outdoor physical activities venues and induces healthy interaction between the human body and the microbial population in the natural ecology. However, the current research is still in its early stages, and the intervention effect and mechanism of pro-nature physical activities need further demonstration in the future.

## 1. Introduction

Psychological stress, defined as a state of mental or emotional tension induced by external stimuli, is widespread in people’s daily lives and can be seen as a challenge or threat that disrupts the body’s internal homeostasis, such as public fear of a new epidemic or adolescent concerns about academic performance. It can cause adverse emotions such as anxiety and depression, and may also lead to a decrease in the body’s immunity and increased susceptibility to disease, or even induce illnesses such as post-traumatic stress syndrome and irritable bowel syndrome (Karl et al., 2018). Because of the prevalence of psychological stress and the complexity of treating its triggers, it has become one of the key research questions in the field of public health worldwide (De Palma et al., 2014).

Physical activity has shown promise as an intervention to improve immunity, and mood states and antagonize psychological stress (Lou and Yan, 2020). However, not all forms of physical activity have antagonistic effects on psychological stress, and inappropriate physical activity can be a source of stress and exacerbate the stress response (Clark and Mach, 2016). Although the characteristics of physical activity interventions in terms of intensity, frequency and duration have been extensively explored, there is no consensus (Biddle et al., 2018) and there is still a need to explore the appropriate forms of physical activity and their mechanisms of action.

Physical activity that integrates contact with nature is known as a pro-nature physical activity, also known as a green physical activity, and is an effective way for humans to engage with the natural environment in modern society, which is important for maintaining the complex symbiosis between humans, microorganisms, and the Earth’s environment, and has shown good value in health management and chronic disease treatment in recent years (Robinson et al., 2018). The American Public Health Association recommends that public health, urban planning, parks, and recreation authorities increase green space for outdoor activity facilities to encourage people to participate in pro-nature physical activities (Hunter et al., 2015). In health care and chronic disease treatment research, the concepts of ‘green prescriptions’ (Ulmer et al., 2016) and ‘park prescriptions’ (Razani et al., 2018) are beginning to emerge, encouraging patients to improve their health by engaging in physical activities in natural environments. With changes in production and lifestyle, humans are spending less time in the natural environment, resulting in reduced exposure to biodiversity, leading to depression, anxiety, and other psychological stress symptoms (Pearson and Craig, 2014), and increasing pro-nature activities are becoming a call of the times. Therefore, pro-nature physical activities are expected to provide a new intervention to combat psychological stress (Wooller et al., 2018).

This study attempts to introduce pro-nature physical activity in psychological stress interventions, to investigate the dual effects of the natural environment and physical activity on psychological stress, to investigate the influence pathways of the environment and physical activity, respectively, based on the microbial-gut-brain axis, and to clarify the possible mechanisms of pro-nature physical activities against psychological stress, to provide a theoretical basis and practical insights for future physical activity interventions to alleviate psychological stress-related disorders.

## 2. Physical activity, psychological stress, and the microbe-gut-brain axis

Roth and Holmes (1987) were the first to investigate the types of exercise used to alleviate psychological stress and concluded that aerobic exercise was an effective intervention. However, subsequent research has been inconsistent, with some studies suggesting that long-term, regular, moderate-to-medium intensity physical activities, such as yoga are more suitable for reducing psychological stress (von Haaren et al., 2015); others suggesting that high-intensity, competitive, and intense confrontational physical activities are an effective stimulus for nurturing the body to adapt to psychological stress (Biddle et al., 2018). Yang et al. (2014) concluded that jogging or basketball at 60–80% of the maximum heart rate, for 45 min each time, twice a week for 12 weeks is a necessary and effective means of alleviating psychological stress in adolescents. From the current study, there is variability in the effects of physical activity to reduce psychological stress, and appropriate physical activity can effectively antagonize psychological stress, but there is still some room for research to optimize physical activity intervention programs.

In terms of explanatory mechanisms, Sothmann et al. (1996) first proposed the “cross-stressor adaptation hypothesis,” which suggests that physical activity induces a series of physiological and psychological adaptations that improve the body’s ability to respond to other stimuli. With the development of brain imaging techniques, Esch and Stefano (2010) found that physical activity can affect the reward and motivation circuits of the nervous system, resulting in self-regulatory and endogenous stress relief. From a psychoneuroimmunological perspective, Yan et al. (2018) and Chen et al. (2009) suggest that physical activity antagonizes psychological stress through physiological change pathways such as cortisol and NK. Thus, there is currently no clear understanding of the explanatory mechanisms based either on altering indicators such as endocrine and immune or on effects on brain function, but it is unclear whether there is a holistic and systematic association between the two in explaining physical activities antagonism to psychological stress.

Recent research has revealed that the Microbiome-Gut-Brain Axis (MGBA) is closely related to host endocytosis (Allen et al., 2017), providing a new perspective on the overall explanatory mechanisms of antagonistic psychological stress in physical activity. The gut microbiota is capable of producing a large number of neurotransmitters (Cryan and Dinan, 2012; Nicholson et al., 2012), while neurotransmitters influence hypothalamic function and the host’s major neuroendocrine axis. A study showing that the gut microbiome is connection with the host neurophysiological systems. The proper functioning of the gut sensory neurons in the Enteric Nervous System myenteric nerve plexus relies on the normal commensal microbiota (Neufeld et al., 2011). Therefore, the MGBA is a bidirectional channel through which the gut microbiota can influence brain function through neural, endocrine, and immune pathways, while the brain can also influence the gut microbiota through neural, endocrine, and immune pathways (Cussotto et al., 2018). The “old friend hypothesis” suggests that the gut has been symbiotic with approximately 100 trillion microbes over thousands of years of human evolution and that the microbiota may be a potential target for the alleviation of psychological stress due to gut-brain interactions (Rook et al., 2013; Evrensel and Ceylan, 2015). The gut microbiota is primarily influenced by genetics, age, and dietary habits, but intervention practices to alter the microbiota through these influences have been slow to progress. In recent years, attempts to influence the diversity, abundance, and composition of host gut microbes through natural environmental exposure and physical activity have shown some promise (Sobko et al., 2020; Tzemah Shahar et al., 2020). Based on the strong association between physical activity, the natural environment and MGBA, and psychological stress, the next step is to explore how the natural environment and physical activity both antagonize psychological stress through MGBA.

## 3. Mechanisms by which pro-nature physical activity antagonizes psychological stress through MGBA

### 3.1. The cyclical association between MGBA and psychological stress

The gut microbiota regulates brain development and function through neurological, neuroimmune and neuroendocrine pathways, and conversely, the brain interacts with microbes through these pathways (Rhee et al., 2009). This two-way communication system is known as MGBA, where signals from the brain can influence intestinal dynamics, secretion, and immune function, while information from the gut can also influence reflex regulation and emotional state in the brain (Huang et al., 2019). The neuroanatomical basis for bidirectional communication in MGBA includes intrinsic neural distribution provided by the enteric nervous system (myenteric plexus and submucosal plexus neurons) and extrinsic neural distribution provided by the autonomic nervous system (enteroendocrine, immune system and microbiota) (Pigrau et al., 2016).

The microbiota in the gut plays an important role in regulating the immune system, influencing host resistance to pathogenic bacteria (Nicholson et al., 2012) and may regulate brain activity by influencing neurotransmitter metabolism (Asano et al., 2012). The microbiota can receive signals from the brain to produce neurochemicals that regulate gut, cognitive and behavioral functions (Lyte, 2014). Hormones and neurotransmitters in the gut are important sources of metabolic signals that regulate the vagus nerve and brain through the enteric nervous system (Field et al., 2010). The neural pathways that signal to the gut from the amygdala and hypothalamus include the HPA axis, sympathoadrenal axis, autonomic nervous system, and monoaminergic pathways (Omran and Aziz, 2014). In contrast, the anterior cingulate cortex and medial prefrontal cortex integrate information about homeostasis and pain in the gut (Ongür and Price, 2000).

The psychological stress response is mostly adaptive, with the body able to restore endocytosis relatively quickly, but as the intensity and duration of the stress increases, the body’s ability to adapt is exceeded and maladaptation occurs. There is growing evidence that the host response to psychological stress is closely related to MGBA. The gut microbiota can influence symptoms of psychological stress such as anxiety and depression (Luczynski et al., 2016). Zhang et al. (2016) showed that psychological stress groups and controls did not show different symptoms of psychological stress, suggesting that the gut microbiota is closely associated with psychological stress symptoms such as anxiety and depression. Psychological stress affects the composition of the gut microbiota and even triggers changes in gut structure (Lobo et al., 2023). Bailey et al. (2011) demonstrated that exposure to psychological stressors affected the levels of cytokines such as interleukin-6 and monocyte chemotactic protein-1 in the gut microbiota. Changes in the microbiota caused by psychological stress may enhance the ability of intestinal pathogens to colonize the gut. Studies in the field of inflammation have also found that psychological stress affects changes in IgA levels in the gut, leading to an imbalance in homeostasis in the gut and an inflammatory response (Campos-Rodríguez et al., 2013). Psychological stress activates the HPA axis, which induces pro-adrenocorticotropic hormones into the body’s circulation and stimulates glucocorticoid synthesis in the adrenal cortex (Smith and Vale, 2006). Catecholamines (norepinephrine and epinephrine) are also released into the somatic circulatory system following psychological stress and, together with glucocorticoids, affect the inflammatory response, with further effects on the brain as the balance between pro-and anti-inflammatory cytokines are disrupted (Lyte, 2011).

In summary, the current evidence demonstrates a circular association between MGBA and psychological stress. On the one hand, psychological stress affects the gut microbiota through the brain-gut pathway; on the other hand, the gut microbiota also affects psychological stress responses such as brain cognition and function through the gut-brain pathway. Therefore, interventions that influence the development of MGBA may be valuable tools to antagonize psychological stress.

### 3.2. Effects of physical activity on MGBA, psychological stress

Physical activity can affect MGBA as well as psychological stress, but different physical activities have opposite effects (Ticinesi et al., 2019). A review by Clark and Mach (2016) on the effects of physical activity on MGBA and psychological stress showed that instead of alleviating psychological stress, prolonged vigorous physical activity (VO_2 max_ ≥ 60–70%) induces a typical stress response. They found that prolonged vigorous physical activity led to increased concentrations of cortisol, epinephrine, and norepinephrine, which reduced blood flow in the viscera and MGBA to redistribute oxygen to the working muscles. The consequent reduction in blood supply to the intestinal epithelium and reperfusion can lead to hypoxia, acidosis, ATP depletion, free radical formation and oxidative/nitrosative stress, which together disrupt the intestinal barrier and lead to increased intestinal permeability. Subsequently, LPS/endotoxin translocation into the circulatory system leads to an inadequate supply of blood, nutrients, water, and oxygen to the intestine, inducing typical stress symptoms such as inflammatory responses and gastrointestinal discomfort.

Although physical activity in the above studies caused adverse reactions to MGBA and psychological stress, however, a growing body of research believes that appropriate physical activity can improve MGBA and psychological stress (Monda et al., 2017). Medium and low-intensity physical activity can influence the gut to shorten the instantaneous bowel movement time, thus reducing the contact time of pathogens with the mucus layer of the gut (Bermon et al., 2015). Therefore, moderate-and low-intensity physical activity has a protective effect on MGBA and can reduce the risk of colon cancer and inflammatory bowel disease (Peters et al., 2001). Under psychological stress, plasma cells and lymphocytes infiltrate the intestine, leading to an increase in villi width, and moderate-and low-intensity physical activity inhibit these morphological changes by reducing cyclooxygenase-2 expression in the proximal and distal intestine, which can act to antagonize inflammatory infiltration and protect the morphology and integrity of the intestine (Campbell et al., 2016). In addition, Matsumoto et al. (2008) found that active participation in running affected microbiota changes and increased n-butyrate concentrations and cecum diameter. As n-butyrate can influence cellular NFB activation and prevent colon cancer and inflammation, appropriate physical activity may reduce the risk of enteropathy by affecting n-butyrate.

Regular physical activity triggers physiological adaptations that can maintain intestinal blood flow during physical activity and reduce the inflammatory response, thereby attenuating physical activity-induced intestinal dysfunction (Lambert et al., 2015). Regular physical activity can regulate gastrointestinal motility through the microbiota (Roager et al., 2016). A study of rugby players found that athletes had a richer diversity of thick-walled phyla than the general population (Clarke et al., 2014). In another study, athletes were found to have more mucus-degrading bacteria in the mucus layer, which were negatively associated with BMI, obesity, and metabolic impairment, and could improve intestinal barrier function (Everard et al., 2013). Furthermore, athletes exhibit lower inflammation, better metabolic markers, and a greater incidence of lower chronic inflammation compared to controls (Handschin and Spiegelman, 2008). However, it is important to note that while regular physical activity can improve immune function, when physical activity is performed again when recovery is inadequate then immune function can be suppressed (Schwellnus et al., 2016).

There are differences in the effects of physical activity on MGBA and psychological stress in different populations. Compared to adults, adolescent physical activities can alter more microbial species and produce more cognitive and psychosomatic effects (Mika et al., 2015). Physical activity during adolescence not only stimulates microbiota development and influences adaptive changes in host metabolism, but also facilitates the healthy development of adolescent brain function (Stilling et al., 2015). In addition, Hong et al. (2003) found that physical activity during adolescence is an effective way to prevent neurodegenerative diseases in old age.

Thus, appropriate physical activity has a beneficial effect on both MGBA and psychological stress, whereas if physical activity is perceived as a stressor, then it can disrupt MGBA and exacerbate psychological stress responses. The current evidence suggests that physical activities that are engaging, low-intensity, enjoyable and regular can counteract psychological stress, particularly in adolescent groups. However, forced participation, high intensity, prolonged and irregular physical activity may be a source of stress and exacerbate the stress response.

### 3.3. The impact of the natural environment on MGBA and psychological stress

In an era of industrialization, urbanization, and the internet, it has been found that ‘grey’ environments lead to a reduction in microbial diversity and induce adverse reactions such as psychological stress (Logan, 2015). The potential value of proximity to the natural environment in alleviating psychological stress has been increasingly appreciated, as the distance from the natural environment may be a contributing factor to many physical and psychological problems. Results have been published both defining natural environments as nature reserves and areas such as parks, greenery, and urban rivers in urban environments, and have found that proximity to these natural environments can reduce psychological stress, improve cognition, improve socialization, and promote physical and mental health (Berto, 2014; Logan et al., 2015; Triguero-Mas et al., 2015). Cohen-Cline et al. (2015) in a twin investigation and comparison found that higher levels of natural environment exposure were associated with a lower risk of depression and anxiety. In contrast, Alcock et al. (2014) found a significant improvement in psychological stress after moving to a more green area through a 3-year follow-up study. Research in the field of ecology also suggests that prolonged residence in a concrete ‘grey environment’ is detrimental to psychological stress responses and that increased green space in cities is associated with increased treatment rates for anxiety and mood disorders (Nutsford et al., 2013). Roe et al. (2013) further confirmed that the natural environment as an intervention can antagonize psychological stress through biochemical indicator tests and questionnaires, finding that greater exposure to nature was associated with healthier salivary cortisol secretion and lower perceived stress.

The gut microbiota is influenced not only by personal characteristics and lifestyle habits but also by the microorganisms in the surrounding environment to which it is exposed (Escobar et al., 2014). The level of green space and vegetation diversity in areas where people are active is associated with the diversity of the human microbiota and can influence the production of allergic IgE responses to common allergens (Ruokolainen et al., 2015). Selway et al. (2020) exposed subjects to the natural environment of an urban park and found that the microbial diversity of the subjects increased and that their composition structure was similar to that of the environment they were exposed to. The microbial structure was somewhat similar to that of the exposed environment. The biodiversity of the environment is associated with psychological stress, possibly through MGBA (Fuller et al., 2007), and Roslund et al. (2020) found that microbial diversity in the natural environment can lead to more diverse microbial associations in children, as well as reduced stress responses, among other effects.

Therefore, exposure to nature can have a positive effect on MGBA and psychological stress, especially for contemporary urban dwellers living in a ‘grey environment’, where pro-nature activities are a natural remedy for many ‘modern diseases’ and are important for the maintenance of both human and natural ecosystems. It is also important for the maintenance of both human and natural ecosystems.

### 3.4. Possible mechanisms by which pro-nature physical activity antagonizes psychological stress through MGBA

Although appropriate physical activity is significantly and positively associated with psychological stress relief, exposure to the natural environment has an effect independent of physical activity, providing additional health benefits to those exposed. A study by Berman et al. (2008) found that subjects walking in forests had higher health benefits compared to walking on urban pavements. De Vries et al. (2013) suggest that pro-nature physical activity mediates the effect of green space distance on psychological stress, whereas total physical activity does not have this mediating effect. Pro-nature physical activity is the perfect form of combining biodiversity contact and physical activity participation, and refers to both physical activities that people engage in to get close to and return to nature, using air, sunlight, water and other health-friendly natural resources, including pro-water (e.g., surfing, diving, etc.), pro-green (e.g., forest bathing, forest exploration) and pro-earth (e.g., mountaineering, orienteering) categories (Editorial Board of the DACIHAI, 2015), but also refers to physical activities in urban environments in areas such as parks, green spaces and urban rivers. Exposure to the natural environment and physical activities are effective means of psychological stress, while available evidence suggests that the combination of these two means may have a superimposed effect (Pasanen et al., 2014; Aspinall et al., 2015).

Pro-nature physical activity may increase microbiota diversity and increase the number of genera of thick-walled bacteria, resulting in greater production of short-chain fatty acids; pro-nature physical activity may also increase levels of Akkermansia bacteria, thereby alleviating metabolic or neurological-related psychological stress responses. Both appropriate physical activity and diverse microbiota exposure can alleviate psychological stress-related irritable bowel syndrome, stabilize the intestinal barrier, reduce anxiety and depression, promote neurotrophic factor secretion, improve the HPA axis, and maintain autonomic nervous system stability (Mackenzie and Brymer, 2018). It is important to note that the current mechanisms are not fully empirical, with pro-nature physical activity antagonizing psychological stress and microbiota alleviation being linked to both, but the extent to which pro-nature physical activity affects the brain through the microbiota and thus alleviates psychological stress disorders needs to be demonstrated.

Pro-nature physical activity antagonizes psychological stress not only in the microbiota of the MGBA, the gut and the brain, respectively, but also in the bidirectional channels of communication between the MGBA. Pro-nature physical activity can antagonize psychological stress, possibly mediated through the vagus nerve. As mentioned earlier, alterations in the microbiota can affect the bidirectional communication of the vagus nerve between the gut and the brain, e.g., bifidobacteria, and lactobacilli. If the vagus nerve is disconnected, the probiotic-mediated effects on the HPA axis and psychological stress are lost (Bravo et al., 2011). Thus, pro-natural physical activity antagonizes psychological stress probably by affecting the vagal pathway between the gut and brain.

The antagonism of psychological stress by pro-natural physical activity may be regulated through BDNF, which plays a key role in alleviating negative emotions such as depression and anxiety. Linnarsson et al. (1997) found that genetic deletion of BDNF leads to apoptosis in mice, and thus is thought to have a cerebral protective effect. And psychological stress symptoms such as irritable bowel syndrome, anxiety and depression are associated with reduced levels of BDNF in the brain (Sarkar et al., 2016). Both oral Bifidobacterium and physical activity can increase BDNF expression, improve nutrient supply to the nervous system, promote neuronal survival, regeneration, and differentiation, enhance neuroprotection and alleviate psychological stress (Sudo et al., 2004; Smith et al., 2010).

The HPA axis is activated when physical activity exceeds 60% of maximum oxygen uptake (VO_2 max_) or during prolonged exercise training, and disrupts the gut microbiota. In athletic competition, athletes are required to perform high-intensity, prolonged physical activity under stress, which also increases HPA axis activation and intestinal disturbances (Lou, 2019). Forcing subjects to perform physical activities may be seen as a psychological stressor that produces similar HPA axis effects to the competition stress athletes face (Cook et al., 2013). An overreactive HPA axis is associated with the microbial imbalance and leads to a psychological stress response, which can be reversed by Bifidobacterium supplementation (Messaoudi et al., 2011), while both moderate physical activity and pro-nature environments can increase Bifidobacterium numbers (Queipo-Ortuño et al., 2013). Thus, pro-nature physical activity affects MGBA, possibly through bidirectional communication in the HPA axis.

An additional pathway of influence may be related to serotonin. Pro-nature physical activities may act to alleviate symptoms of depression and anxiety by increasing the synthesis and metabolism of 5-hydroxytryptamine (Wipfli et al., 2011). At the same time, some intestinal microorganisms such as Lactobacillus spp. of the thick-walled phylum can also produce serotonin (Özoğul, 2004; Özoğul et al., 2012). Pro-nature physical activities may support the increase of these strains by influencing the diversity of the gut microbiota. Thus, serotonin production through MGBA compatible with the pro-nature physical activity may be one of the pathways explaining participants having lower psychological stress symptoms.

**Figure 1:**
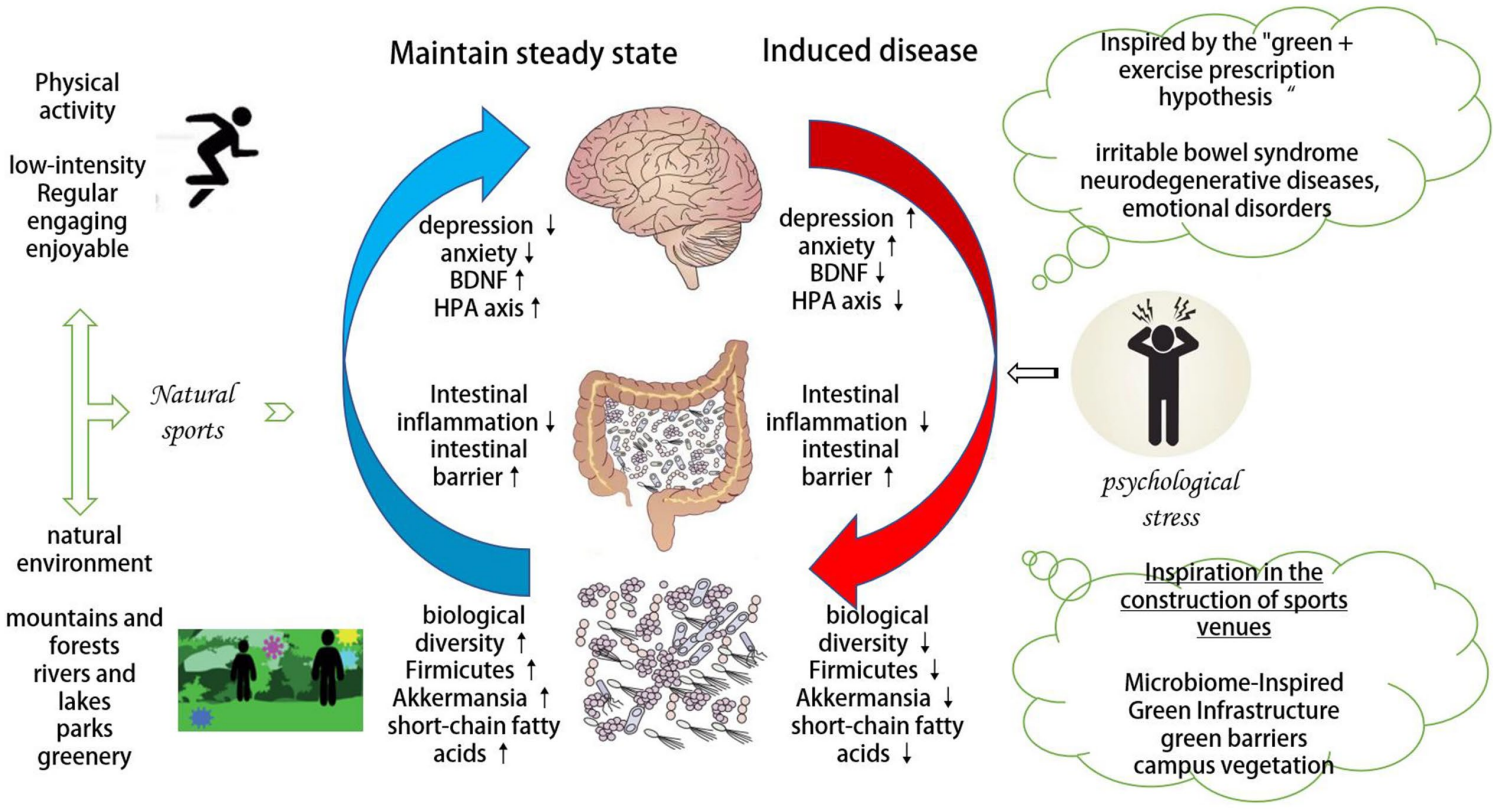
Mechanisms by which pro-nature physical activity antagonizes psychological stress through MGBA.

In summary, pro-nature physical activity can antagonize psychological stress by a mechanism related to MGBA (Figure 1). However, the extent to which MGBA mediates the antagonism of psychological stress by pro-nature physical activity, whether there is a superimposed effect, and how the two-way communication mechanism operates, although hypotheses are made theoretically in this paper, need to be tested empirically in the future.

## 4. Inspiration for the application of pro-nature physical activity to alleviate psychological stress through MGBA

### 4.1. Inspiration for exercise interventions for psychological stress-related conditions

Psychological stress leads to dysregulation of MGBA, causing decreased biodiversity of the microbiota, loss of beneficial metabolically active symbionts, and overgrowth of pathogenic bacteria (Mosca et al., 2016; Levy et al., 2017; Kriss et al., 2018). The balance between group and host physiology is disrupted (Buttó et al., 2015), and intestinal permeability increases, causing bacteria or bacterial toxins and metabolites to enter the systemic circulation, which promotes inflammation and related diseases (Logan et al., 2016). Based on the above discussion, an intervention based on pro-nature physical activity can be proposed, which is called “green + exercise prescription.” Because it can affect psychological stress through MGBA, it is expected to solve psychological stress-related diseases such as irritable bowel syndrome, neurodegenerative diseases, and emotional disorders.

With the changing environment of modern life and increased competitive pressures, irritable bowel syndrome has become a highly prevalent psychological stress-related intestinal dysfunctional disorder with a global prevalence of 11.2% and a prevalence of 12.4% in China, and a *per capita* treatment cost of tens of thousands of yuan (Lovell and Ford, 2012; Zhang et al., 2016; Bai et al., 2017). Current pharmacological treatments cannot cure the disease and may produce adverse side effects (Camilleri and Ford, 2017). Pro-nature physical activity has the potential to provide new non-pharmaceutical treatments for irritable bowel syndrome. Irritable bowel syndrome is associated with abnormal gastrointestinal dynamics, gut-brain dysregulation, microbiota dysbiosis, gastrointestinal inflammation, and central nervous system dysregulation (Zeng et al., 2020). As discussed previously, pro-nature physical activities can regulate MGBA, stabilize the microbiota, improve gut health, and regulate brain function. Zhao et al. (2013) used a mountaineering intervention in 73 patients with irritable bowel syndrome and found that after 6 weeks, mountaineering significantly improved abdominal pain, bloating, diarrhea, and constipation, and was more effective than the oral trimebutine maleate medication group. They concluded that mountain climbing is a fun and recreational physical activity that stimulates gastrointestinal motility, improves digestive function, and promotes *β*-endorphin secretion and that the proximity to nature during mountain climbing is beneficial in relieving individual psychological stress. In a study by Johannesson et al. (2011), it was found that voluntary outdoor exercise such as low to moderate intensity walking, jogging, swimming, and cycling significantly improved GI symptoms in patients with irritable bowel syndrome. At 5 years follow-up, it was found that consistent participation in the exercise had a long-term positive effect on GI symptoms, depression, and anxiety (Johannesson et al., 2015). Therefore, for patients with irritable bowel syndrome, due to the complex link between its pathogenesis and MGBA and psychological stress, then the pro-nature physical activity is expected to provide an effective integrated systemic intervention because of its effectiveness in improving MGBA and psychological stress status.

Psychological stress is associated with a variety of neurodegenerative diseases, such as Alzheimer’s disease, Parkinson’s syndrome, and Huntington’s chorea (Gubert et al., 2020). There is growing evidence that MGBA plays a key role in the mechanisms of the onset and progression of Alzheimer’s disease (Wu et al., 2017), Parkinson’s syndrome (Bedarf et al., 2017), and Huntington’s chorea (Kong et al., 2020). Physical activity can modulate cognitive function and improve neurodegenerative diseases by establishing a gut-brain link through the modulation of gut microbes, but there is variability between the effects of different modalities of an exercise intervention on gut microbes and their modulation with cognitive function (Yu et al., 2022). As pro-nature physical activity can antagonize psychological stress through MGBA, it has potential applications for the treatment and rehabilitation of neurodegenerative diseases. Hong et al. (2003) investigated 127 cases of Alzheimer’s disease and 127 case-matched samples and found that outdoor physical activity at a young age was an effective means of preventing Alzheimer’s disease in old age. Ma et al. (2022) analyzed 9-year follow-up data from a United Kingdom Biosample cohort of nearly 370,000 samples and found that outdoor activities play an important role in actively intervening, preventing and slowing the occurrence and development of Alzheimer’s disease, which they suggest may be related to the ability of outdoor physical activity to act as a neuroprotective, immune response modulator, inflammation suppressor and regulator of oxidative stress. Thus, the current evidence suggests that pro-nature physical activity not only has a therapeutic effect on patients with neurodegenerative diseases but also that pro-nature physical activity at a young age may be an important means of preventing or delaying the onset of neurodegenerative diseases.

Mental stress-induced depression, bipolar disorder and other mood disorders are associated with MGBA dysregulation. Xue et al. (2019) suggested that appropriate exercise could improve the abnormal expression of peripheral or central brain gut peptides, which in turn affects monoamine neurotransmitter levels, HPA axis activity, neurotrophic factor expression and neuroplasticity, thus acting as an antagonist to depression. Wang and Wang (2018) concluded from a survey of 386 university students that being active in a natural environment significantly and negatively predicted depressed mood. Xu et al. (2022) conducted a study on the relationship between living arrangements and depressive symptoms in 12,200 elderly people, and found that outdoor physical activities had a good effect on reducing depressive symptoms in the elderly. Bipolar disorder has a similar high abundance of actinomycetes, enterobacteria and low abundance of faecal bacteria as depressed patients, and appropriate physical activity has also been prescribed as a treatment for bipolar disorder (Khoubaeva et al., 2022). In conjunction with the aforementioned suggestion, that appropriate physical activity and biodiversity exposure can influence MGBA to antagonize psychological stress, pro-nature physical activity may be an effective means of treating mood disorders to help restore balance to the gut microbiota system in organisms suffering from mood disorders.

Due to the complex pathogenesis of psychological stress-related disorders, which are often triggered by multidimensional causes, treatment can be multi-targeted and relapse-prone, and currently, pharmacological treatments are inadequate in terms of efficacy and side effects. The ability of pro-nature physical activity to antagonize psychological stress through MGBA can intervene in the organism as a whole and is expected to provide a new therapeutic or complementary treatment for the alleviation of psychological stress. It has been suggested that for some chronic diseases that are currently incurable, the future philosophy of healing may move toward a holistic and integrated view, replacing the fine-grained view of drugs (Fan, 2017). Pro-nature physical activity has the potential to be such a holistic therapy by antagonizing symptoms of psychological stress through MGBA, however, this idea is still in the early stages of research and therefore there is scope to explore the efficacy and mechanisms of using pro-nature physical activity for psychological stress-induced disorders in the future.

### 4.2. Inspiration in the construction of outdoor physical activity venues

Based on pro-nature physical activity mediating MGBA to antagonize psychological stress, outdoor physical activity relying on mountains, water, grasslands, and other pro-nature activities are advocated. However, for modern people living in urban areas, it is also possible to build parks with physical activity venues near where they live, work and study; or when building green paths and courts for residents to exercise, consider avoiding direct connection with grey buildings and roads, and can build green belts and small gardens for partitioning, creating local spaces of multi-biological nature. This is in line with the Microbiome-Inspired Green Infrastructure (MIGI) advocated by Robinson et al. (2018), which is a system engineering approach that refers to living, multifunctional green spaces designed and built to It is designed and built to induce interactions between the human microbiome and the natural ecology of the microbiota. According to the theory of microbial rewilding, biodiversity can be restored in small spaces of microbial habitat when MIGI is constructed in outdoor physical activity venues in cities (Mills et al., 2017). Therefore, physical activity in such urban green spaces may obtain similar MGBA impacts to those of mountains and forests.

The construction of green barriers for outdoor physical activity venues may be effective, such as hedges of trees and grasses on either side of fitness trails, or fences of greenery at outdoor fitness equipment sites. For cage football and basketball courts with iron nets, vines can be planted to form a green barrier. Natural green walls at physical activity venues can increase biodiversity exposure, reduce noise pollution, improve the sensory experience, and reduce pollution by trapping airborne dust and particulate matter (Van Renterghem et al., 2013; Abhijith et al., 2017). Green barriers help protect humans and microbial communities in green spaces from industrial pollutants and help reduce respiratory diseases (Soyiri and Alcock, 2018).

Given that pro-nature physical activities may have a beneficial effect on MGBA, growth and development, and physical and mental health of adolescents, the amount of time adolescents spend outdoors in contact with nature in modern society has drastically decreased (Soga et al., 2016). To facilitate young people’s exposure to biodiverse natural environments, schools that are in a position to do so should encourage outdoor activity programs that are mountain-friendly, water-friendly and green-friendly. At the same time, greenery and vegetation around activity venues should be considered in school construction, and microbial-inspired infrastructure based on the idea of creating small ecologies in cities is needed to encourage young people to engage with nature, increase pro-nature physical activities, and reduce screen time and sedentary time (van Sluijs et al., 2021).

## 5. Conclusion

There is a circular association between MGBA and psychological stress. Psychological stress can affect the microbiota through the gut-brain pathway, and conversely, the microbiota can also affect psychological stress-induced symptoms. Inappropriate physical activities methods and environments can cause MGBA disorders and aggravate the adverse reactions of psychological stress. It is suggested that the microbe-gut-brain axis may provide a new perspective and target for the treatment of psychological stress-related diseases. Pro-nature physical activity can improve the number of Firmicutes, short-chain fatty acids, Akkermansia bacteria, and the gut-brain barrier and further affect the HPA axis, BDNF, and serotonin pathways of gut-brain two-way communication, thereby maintaining the body’s homeostasis and reducing antagonistic psychological stress. This article further puts forward the “green + exercise prescription hypothesis.” Hypothesis of the holistic medical view, which is expected to provide new means in the prevention, alleviation, and treatment of irritable bowel syndrome, neurodegenerative diseases, emotional disorders, and other psychological stress-related diseases. In addition, this review article also proposes the construction of green infrastructure that is conducive to the diverse contact of microorganisms in outdoor physical activities and physical activity venues and induces the interaction between human microbiota and microbiota in natural ecology.

However, the current research is still in its infancy, and the intervention effect and mechanism of pro-nature physical activity need further demonstration in the future. More research is also needed to understand whether there is an optimal program of pro-nature physical activity for different symptoms, such as exposure to the degree, time, type, form, intensity, frequency, etc. of exercise. Physical activities are not only beneficial to human beings but also have the potential to bring common benefits to the natural environment and are also meaningful to the protection of biodiversity on the planet. Pro-nature physical activity may help preserve and restore microbial habitats in the human body and in the natural environment, rewild the microbiota, protect “old friends” that have coexisted with humans for thousands of years, and promote health through various benefits from the interaction.

## Author contributions

HL: study design, paper writing, and paper revising. XL: literature search and summary of views. PL: paper revising and integral design. All authors contributed to the final manuscript writing and revisions.

## Funding

This work was supported by General program of Education of the National Social Science Fund of China: “Research on activities regulation mechanism and intervention scheme of middle school students’ psychological pressure” (BLA210215).

## Conflict of interest

The authors declare that the research was conducted in the absence of any commercial or financial relationships that could be construed as a potential conflict of interest.

## Publisher’s note

All claims expressed in this article are solely those of the authors and do not necessarily represent those of their affiliated organizations, or those of the publisher, the editors and the reviewers. Any product that may be evaluated in this article, or claim that may be made by its manufacturer, is not guaranteed or endorsed by the publisher.
